# Drug resistance in glioblastoma: are persisters the key to therapy?

**DOI:** 10.20517/cdr.2020.29

**Published:** 2020-08-07

**Authors:** Lisa Oliver, Lisenn Lalier, Céline Salaud, Dominique Heymann, Pierre François Cartron, François M. Vallette

**Affiliations:** ^1^Université de Nantes, INSERM, CRCINA, Nantes 44007, France.; ^2^CHU de Nantes, Nantes 44093, France.; ^3^LaBCT, ICO, Saint Herblain 44805, France.

**Keywords:** Drug resistance, persisters, tolerance, chromatin remodeling, metabolism, tumor environment

## Abstract

Glioblastoma (GBM) represents the main form of brain tumors in adults, and one of the most aggressive cancers overall. The treatment of GBM is a combination of surgery (when possible), chemotherapy (usually Temozolomide, TMZ) and radiotherapy (RT). However, despite this heavy treatment, GBM invariably recur and the median length of survival following diagnosis is 12 to 15 months, with less than 10% of people surviving longer than five years. GBM is extremely resistant to most treatments because of its heterogeneous nature, which is associated with extreme clonal plasticity and the presence of cancer stem cells, refractory to TMZ- and RT-induced cell death. In this review, we explore the mechanisms by which cancer cells, and especially GBM, can acquire resistance to treatment. We describe and discuss the concept of persister/tolerant cells that precede and/or accompany the acquisition of resistance. Persister/tolerant cells are cancer cells that are not eliminated by treatment(s) because of different mechanisms ranging from dormancy/quiescence to senescence. We discuss the possibility of targeting these mechanisms in new therapeutic regimen.

## Introduction

Cancer happens unexpectedly for the most part and by the time it is detected it has already gone through numerous “stop or go” cycles. Although not very well documented, it is assumed that most precancerous cells are eliminated from the body through processes implicating cellular mechanisms (e.g., activation of cell death programs) or global reaction (e.g., immune system control). Hence, at diagnosis, cancer cells are likely to have acquired several mechanisms reinforcing cell survival and/or escaping from immune surveillance. Cellular heterogeneity, a common landmark in many cancers, could be the direct consequence of a pre-diagnosis selection/adaptation process. Most current treatments target proliferation and/or survival pathways in cancer cells and as such could trigger another level of selection and/or adaptation. Therefore, in some cancers, after an initial reduction in tumor mass, surviving resistant cells are detected. Understanding the mechanisms, which lead to the acquisition of resistance by tumor cells is one of the major challenges that the medical and scientific research community need to address to eradicate cancers.

Glioblastoma (GBM) represent the most frequent primitive brain tumors in adults. Their current treatment - generally described as the protocol “Stupp” - is a combination of complete surgical resection, followed by radiotherapy and concomitant chemotherapy with the methylating agent Temozolomide (TMZ)^[1]^. Since its introduction, this regimen has had a deep impact both on overall survival and on progression-free survival^[1]^. However, since then, no new treatment has been discovered and the median survival time (circa 15 month) has not been increased over past 20 years. The quest for a more effective therapy remains a primary aim in the GBM community. The reason of this lack of progress is linked to the complexity of GBM, which is extremely heterogeneous by nature (the original name of GBM was Glioblastoma Multiforme, a name that speaks for itself) and/or with an extraordinary plasticity. In addition, evidence suggests that GBM contains a population of cancer stem cells that are highly resistant to current therapies.

## General consideration on cancer resistance to treatment

### Most common cellular mechanisms

**Figure 1:**
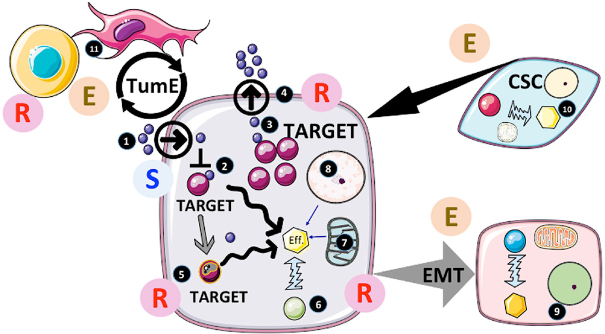
Actors and factors implicated in cancer resistance to treatments. 1. The accessibility of the drug to cancer cells is usually determined during clinical assays (pharmacokinetics, pharmacodynamics…). In the case of intracellular targeting, the transit of the drug across the plasma membrane can also be critical and is usually tested during pre-clinical studies. However, the drug distribution can be affected by both elements in the tumor microenvironment (TME) as well as by changes in the body response, which will result in resistance by forfeit; 2. If the drug is delivered and affects the activity of the target, several factors could intervene modifying the structure of the drug and/or its delivery to another compartment (e.g., endosomes) thereby decreasing its efficiency. If the target is upstream of a major cellular effector (Eff.) responsible for cancer progression, other pathways can be activated to sustain this activity. This would result in circumventing the treatment efficacy and induce escape; 3. Overexpression of the target would also limit the efficiency of the inhibitor and trigger resistance if an increased dose (due to side-effects) is not possible; 4. If the drugs were substrates of the multidrug resistance (MDR) pumps, activation of MDR would limit the intracellular concentration and impede its therapeutic activity; 5. One of the most-documented resistance mechanisms is mutation of the target as often observed with tyrosine kinase inhibitors (TKI) such as EGFRi or BRAFi. *Per se*, mutated cells insensitive to treatment slowly emerge from the treated tumor mass and become predominant after an initial tumor regression. Of note, the process implicated is still not completely understood (preexisting clones, induced mutations or selected random mutations); 6. Another common process observed with TKI is the bypassing of the target for the activation of the effector. Often another tyrosine kinase pathway can be amplified to compensate for the inhibition of the target. In addition, deep modifications of the cellular phenotype/genotype induced by the treatment directly or indirectly can also contribute to resistance; 7. Mitochondria are central to resistance through two distinct processes. First by rewiring metabolism; cancer cells can adapt to the inhibitory effects on target under many circumstances (providing alternative sources of core elements for the cells to proliferate such as nucleic acids, amino acids, ATP sources…) or epigenetic modifiers (see below). Secondly, mitochondria are central to apoptosis and other cell death programs^[44]^. Consequently, modification of mitochondrial activity can also play an active role in cancer cell survival through the life/death response following the inhibition/modulation of target. In fact, resistance to apoptosis is one of the hallmarks of cancer and plays an important role in in the escape by cancer cell to immune-surveillance; 8. Cancer genome modifications can occur through DNA mutations as mentioned but also through changes in epigenetic regulation such as miRNA production, DNA methylation and histone modifications. In this respect the metabolic input is of major importance providing key co-factors called oncometabolites, which includes alpha keto-glutarate or succinate necessary for the function of epigenetic enzymes. These oncometabolites participate in the cellular reprogramming often observed under the selective pressure induced by treatments^[93]^; 9. Another phenomenon triggered by the treatment is the epithelial-mesenchymal transition (EMT). EMT is a well-known process in the context of pathophysiological conditions, such as repair of injured tissue. EMT leads to the dissolution of cell-cell contacts, morphogenetic changes, increased motility and invasiveness. In cancer, EMT is accompanied by metabolic, epigenetic, and differentiation reprograming, all of which participate in drug resistance^[94]^. In GBM, a transition to mesenchymal phenotype is often observed^[95]^. This EMT-like process provides a selective advantage to cancer cells culminating in an escape from treatment^[96]^; 10. In many tumors (but not all), the existence of cells with some “stem cell” properties (such as stem cells markers, self-renewal, high DNA repair capacity, resistance to cell death) has been shown. Because of the aforementioned properties, cancer stem cells (CSC) have been proposed to be the cornerstone of both cancer heterogeneity and treatment resistance and the main culprit responsible for recurrence^[87]^; 11. Tumor microenvironment (TME) is an essential component of cancer growth and survival. The implication of TME in cancer resistance as well as sensitivity to treatment has been proposed^[96]^. Non-tumor cells present in the microenvironment are multiple and thus a change in TME composition is likely to influence cancer response to therapy in both ways (i.e., escape and resistance). TME can secrete factors or directly provide elements by cell-cell interactions that could participate in tumor growth and acquisition/selection of resistant cells^[96]^. In addition, it should be kept in mind that immune cells are part of TME and that cancer cells usually inhibit their function. The actual importance and role of TME still needs more evaluation but it is clear that it represents a major axis of research for new therapies. E: escape; R: resistance; S: sensitivity to treatment

Several excellent reviews have recently described the current mechanisms of resistance in cancers^[2-8]^. Figure 1 provides an overview of the main mechanisms responsible for the acquisition of resistance to drugs by tumors.

### Cell death in drug resistance

**Figure 2:**
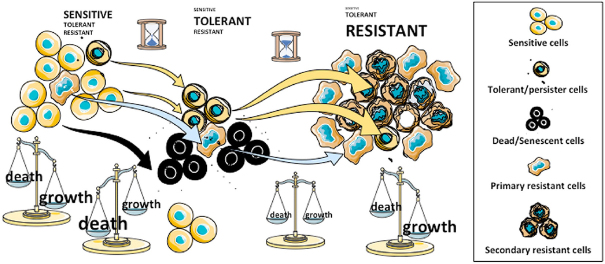
Cell Death and resistance to treatment in cancer. Distinct cell populations and states underline the dynamic and plasticity of emergence of resistant cancer cells. Black dots surrounding dead/senescent cells are death-inducing factors that could support the survival in surrounding cells. Treatments could trigger cell death (or senescence) in tumor cells, subsequently called “sensitive” while the same treatment could be ineffective, not only in the resistant population, be present at a low level but also in persisters (left). In the first stage of treatment when the wave of cell death is over, persisters would be predominant (middle). Resistant clones would arise from the viable pool and with time acquire high proliferation rates (right). Whether or not, some resistant cells will derive from persisters, as a secondary resistant population, is not known. At the end of this process, resistant cells might encompass multiple mechanisms of resistance, which will prevail along with persisters or persister-derived “sensitive” cells (right)

Regulated cell death programs play a central role in the elimination of tumor cells. Figure 2 illustrates the importance of cell death in treatment resistance: the first response of cancer cells to most treatments is usually cell cycle arrest followed by cell death^[9]^. Failure to induce apoptosis, the most common form of the cell death programs, has been observed in many cancers and seems to be co-substantial to this disease^[10]^. Several drugs designed to re-activate cell death are now increasingly used in new regimens in combination with conventional treatments^[9]^. However, due to the complexity of drug resistance and possible side effects, the effectiveness of these treatments is still restricted to only few, mostly hematologic, malignancies. In addition, several forms of cell death can be engaged by tumors and other programs such as autophagy and senescence have also been reported to stop or slow down cancer progression. However, there is a major caveat in the induction of massive cell death as dead cells can produce signals that either protect other cancer cells or trigger the activation of cancer stem cells. Factors implicated in these processes are numerous and not well defined. However, several studies have pointed out that prostaglandin E2 could be an important survival signal for neighboring cancer cells^[11]^. Again, the implication of TME in this process is not well known but it could be decisive for the survival of cancer cells^[12]^. As illustrated in Figure 2, the balance between death and growth in untreated cancer (at diagnosis) is in favor of growth. Treatment is usually designed to kill cancer cells and often, massive cell death occurs shortly after the treatment. A lag period during which it is likely that cells are neither dying nor proliferating follows and precedes the reappearance of fast-growing tumors resistant to the treatment.

### A new population implicated in resistance, the drug-tolerant persister cells

Recently, many groups have identified a subpopulation of cancer cells called “persisters”, which share common properties of drug tolerance with persisters observed in bacteria population that are produced with antibiotic resistance^[13]^. Persisters are normal cells rendered drug-tolerant through reversible, non-mutational mechanisms such as chromatin or metabolic remodeling^[13]^. The putative role of these persisters in drug resistance and tumor progression is described in Figure 2. It has been shown in lung cancer that the drug-tolerant persisters exhibit a repressed chromatin state characterized by increased methylation of histone H3 lysine 9 and 27 (H3K9 and H3K27)^[14]^. Indeed, modifiers of chromatin such as histone lysine demethylase genes (KDM) appear to be the best persister biomarkers to date^[15]^. In the clinical field, however, the description of persisters is still an open question as it is also their relationship with other types of slow cycling minor cancer cell populations such as dormant, quiescent or cancer stem cells^[16]^. However, the distinction between persisters and resistant cells may be their capacity to proliferate under treatment. The origin of persisters is unclear and could be linked to stress, phenotypic plasticity or stochastic cell-to-cell^[15,17]^. Ramirez *et al*.^[18]^ have shown that resistance can arise from gene mutations and slow-growing persisters after long-term treatment.

Persisters were found in many solid tumors originating from different organs (lung, skin, brain, colon…) and under different treatments^[16]^. These findings suggest that persisters with drug-tolerant phenotypes could be a common feature in cancer. Nevertheless, even if the phenotypes of these cells share common traits it is possible that distinct characteristics are conditioned by both the nature of the tumor and the treatment applied; and as such could trigger distinct types of persisters. Despite that, the reversibility of the persister phenotype has been shown in most but not all persisters.

The essential question now is to characterize persisters in cancers and to compare with persisters in bacteria, which could provide some clues. The origin of persisters in bacteria is still under discussion but could be linked to mechanisms ranging from stochastic regulation to an active induced state^[13]^.

1. Persisters could be present in the non-treated population. It has been established that in normal growing bacterial biofilms, persisters might account for about 1% of the population^[19]^. The percentage of persisters is currently not known in most cancers.

2. If persisters were generated through phenotypic fluctuation, they would be generated at each generation and not necessarily immediately eliminated by selection due to their low metabolism, slow cycling, and resistance to cell death. Of note, in bacteria, persisters have been shown to evade immune surveillance, which could provide another selective advantage to them. Whether or not this process is specific to bacteria or a feature of all growing tissues (including cancer) is a pending question. Cancer persisters could be a minor subpopulation present in untreated cancers, possibly exhibiting slow cycling and death [Figure 2].

3. In bacteria, persisters can also be induced by the environment and/or specific signals^[13]^. Indeed, the introduction of antibiotics could be an amplifying signal for the genesis of persisters by a simple selective process (to die or not). Several mechanisms responsible for the induction of persisters have been proposed including change in specific death signals, metabolism, and stress response in bacteria^[20]^. Since cancer treatments trigger numerous stresses one can postulate that they could cause a significant increase in persisters.

4. The antibiotic tolerance of persisters in bacteria has been shown to depend on the amplification of certain proteins that otherwise would trigger cell death^[13]^. It is thus possible that conditions under which certain proteins are produced lead to an increased in persisters through reduction of cell cycling and/or induction of dormancy. Alternatively, it has been proposed that persisters do not rely on any specific mechanisms but are simply the consequences of growth reduction^[20]^. Studies on cancer persisters have shown that metabolic; cell survival and epigenetic changes in persisters are often accompanied by slow cell growth^[16]^.

At this stage, one can hypothesize that persistence and resistance could be two independent responses to treatment and that the cross talk between persisters and resistant cells is necessary to produce the fast-growing resistant populations. Alternatively, it is possible that resistant cells could derive from persisters through transformation/mutation induced by the treatments. However, although a complete and thorough characterization of persisters are still underway: there is evidence of the probability of genetic heterogeneity, which would require the induction of a key number of limited mechanisms to survive. This “bottleneck” might represent a new target for the rational design of efficient lines of therapy to overcome treatment resistance.

## GBM and resistance to treatment

### Mechanisms of resistance specific to TMZ

The resistance to TMZ in GBM has been reviewed in detail by Lee^[21]^. The DNA alkylating drug TMZ is the only drug with therapeutic activity against high-grade GBM and has become a part of the standard treatment of these tumors in combination with radiotherapy^[1]^. TMZ is 100% bioavailable when taken orally and, because of its small size and lipophilic properties, it can cross the blood-brain barrier^[22]^. In cancer cells, TMZ induces a cell cycle arrest at G2/M, which is followed by the induction of apoptosis. At the DNA level, TMZ adds methyl groups at N^7^ and O^6^ on guanine and O^3^ sites on adenine, which trigger different DNA repair pathways. However, The extent of methylation at the O^6^ position of guanine in DNA correlates well with the therapeutic activity as well as the toxicity of TMZ^[23]^. One of the consequences of the guanine methylation is an abnormal pairing with thymine instead of cytosine, which leads to mutations in the absence of efficient base exchange repair and DNA mismatch repair^[23]^. O^6^-methylguanine DNA methyltransferase (MGMT) is a suicide DNA repair enzyme, which demethylates the O^6^ position of guanine and thus counteracts the TMZ effect. About 50% GBM patients benefit from multiple administrations of TMZ and this efficiency correlates with the silencing of MGMT by DNA methylation on its promoter^[24]^. The methylation of the MGMT promoter by small methyl donors such as folate has been shown to silence its expression and consequently to enhance TMZ efficacy in MGMT-expressing GBM^[25-27]^. However, the expression of MGMT can be induced in MGMT negative tumors upon TMZ treatment^[28,29]^. As such, MGMT appears to be the main modulator of TMZ resistance in GBM as expected from its biochemical role.

Another important factor, which has been implicated in TMZ resistance, is p53 but its function has not been clearly established. Most mutations of p53 in GBM are gain of function^[30]^. Indeed, GBM cell lines with non-functional p53 were significantly more sensitive to TMZ^[31]^ while small molecule activators of p53 have been shown to effectively enhanced TMZ effects in GBM xenografts *in vivo*^[32]^. In p53 wild-type cells, TMZ promoted the phosphorylation of p53 at Ser15 and Ser46. Since these two post-translational modifications have opposed functions in survival and death^[33]^, however, their role in TMZ resistance is unclear. Consequently, the implication of p53 in the action of TMZ could be related to its post-translational modification ratio.

Due to its plasticity, epigenetic alterations have been described as crucial drivers in acquired chemo-resistance. The acquired resistance seen during TMZ administration in GBM patients is no exception to this rule. The study of MGMT methylation level in untreated GBM patients is considered as the most relevant epigenetic biomarker associated with the predictive response to conventional treatment^[34]^. Analyses of the MGMT methylation status in primary vs. recurrent GBM showed that TMZ induced modifications in the MGMT gene methylation that promoted MGMT expression in recurrent GBM^[35]^. In addition to methylation of its promoter region, the epigenetic regulation of a MGMT enhancer was also reported as a regulator of treatment resistance in GBM^[36]^. de Souza *et al*.^[37]^ suggested that CpG Island Methylator Phenotypes (CIMP) could be used as predictive biomarkers for recurrence in GBM after TMZ treatment. Belter *et al*.^[31]^ observed a global DNA hypomethylation in the range of therapeutically achieved TMZ concentrations over longer exposure times. Lu *et al*.^[38]^ report that the hypomethylation of promoter region of SNHG12 occurs in TMZ-resistant cells and that this contributed to lnc-RNA-SNHG12 activation resulting in TMZ resistance. Thus, TMZ effect on DNA methylation could be responsible for the increase expression of MGMT in GBM.

Other studies have identified other epigenetic players associated with the TMZ resistance and/or GBM recurrence. For example, Briand *et al*.^[39]^ reported that the TET2 expression increased the between primary and secondary resection in patients treated with the Stupp protocol. Banelli *et al*.^[40]^ reported that the expression of KDM was increased in TMZ-resistant cells compared to TMZ-sensitive cells, and that TMZ-resistance was mimicked by over-expression of KDM5A while TMZ-sensitivity was mimicked by inactivation of KDM5A. These results are interesting, as KDM have been implicated in the persister state in GBM^[41]^.

In addition to the use of tumor resection samples, the use of liquid biopsies appears as a promising alternative to perform longitudinal studies of epigenetic signatures associated with the acquisition of resistance. The MGMT methylation level in blood and cerebrospinal fluid has been shown to be promising^[42]^. In addition, several studies such as that published by Nadaradjane *et al*.^[43]^ suggested that the monitoring of cell-free miRNA in blood could be a real time biomarker associated with acquired TMZ-resistance.

### Death and survival mechanisms in GBM as resistance mechanisms

Apoptosis is the central cell death program regulating cellular homeostasis and much pathology in eukaryotes^[44,45]^. Numerous recent reviews have shown the importance of cell death in GBM and its potential use in clinic^[9,46]^. Apoptosis is the main cell death program and the B-cell Lymphoma-2 (BCL-2) family of proteins is instrumental in the completion of apoptosis (and probably other forms of cell death)^[10]^. The balance between pro- and anti-apoptotic members of the BCL-2 family is the key element in the control of apoptosis^[44]^. This balance between pro- and anti-apoptotic proteins is a landmark of GBM progression^[47,48]^. *In vitro* studies have shown that the inhibition of the protein Bcl-2 sensitized GBM cells to apoptotic inducers^[49]^. Our group has shown that TMZ induced a rapid shift in the dependency of anti-apoptotic members by promoting the degradation of Mcl-1 thereby promoting Bcl-2/Bcl-Xl-induced resistance to apoptosis in GBM cell-lines^[50,51]^. Subsequently, the pharmacological inhibition of anti-apoptotic members of the BCL-2 family could represent a novel strategy for in the treatment of cancer; and small-molecules targeting the BCL-2 family, including ABT-263, can augment GBM elimination when combined with other chemotherapeutic agents^[49]^. Of note, the use of ABT-263 should be evaluated in combination with radiotherapy as it selectively kills senescent cells^[50,51]^. Also, the question of the capacity of inhibitors such as ABT-263 to efficiently cross the blood brain barrier has still to be firmly established^[52]^. Furthermore, the side effects of these drugs on normal cells as well as the use of the proper target (Bcl-2, Bcl-Xl, Mcl-1…) at the appropriate time are also major problems that have to be solved before their therapeutic use.

**Figure 3:**
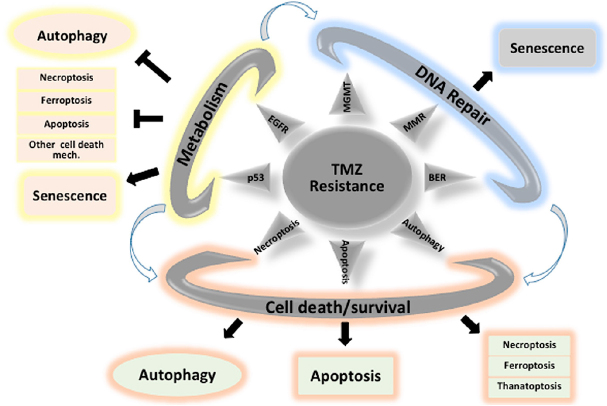
Major mechanisms implicated in TMZ resistance: impact of cell death/survival mechanisms. TMZ: Temozolomide

The mechanisms by which TMZ eliminates GBM cells are still not universally accepted and several mechanisms including apoptosis, senescence and autophagy have been proposed to be associated with TMZ cytotoxicity^[53]^. Autophagy, apoptosis, and senescence could co-exist as stress-induced processes and could probably operate together in a cell autonomous or non-autonomous manner. As described in Figure 3, a failure to induce apoptosis or autophagy might unmask other cell death mechanisms such as necroptosis or ferroptosis^[54,55]^. The level of autophagy induced by TMZ could also depend upon different factors such as the concentration of TMZ, the degree of DNA damage, hypoxic or metabolic conditions or clonal variations^[56]^. The degree of autophagy could lead to adverse situations: too much or too little autophagy leading to death or an intermediate “goldilocks zone” that could promote survival. The complexity of autophagy induction and the role(s) in the GBM could be the reason of this controversial role in GBM and as such hamper therapeutic intervention^[56]^.

Senescence, which can be induced by TMZ^[57]^ and irradiation^[58]^, has become an attractive feature in GBM therapy. Of note, TMZ-induced senescence depends on p21 activation and thus on the presence of wild type p53. In p53-deficient cells, which cannot activate p21, TMZ would not induce senescence^[59]^, which nonetheless can be revealed in case of autophagy deficiency^[60]^.

TMZ-induced senescence was accompanied by an abrogated (suppressed) DNA repair, which included mismatch repair and homologous recombination. Pro-senescence drugs such as inhibitors of CDK4/CDK6, alone or in combination with other treatments are currently under evaluation in clinical trials but the results have not been conclusive so far^[61]^. On the other hand, the impact of senolytic drugs on GBM has not yet been published. TMZ could under certain circumstances activate directly or indirectly cell death mechanisms and/or modify the fine balance between the many mechanisms controlling survival pathways [Figure 3].

## Metabolism and GBM resistance to treatment

GBM cells are highly proliferative and strongly depend on aerobic glycolysis for their survival, which would result in an increase in basal levels of reactive oxygen species (ROS)^[62]^. Both TMZ and radiotherapy induce DNA damage and cell cycle arrest, ROS production and activation of kinase signaling pathways. Aside from the preponderant role of MGMT expression in cell survival, the relationship between GBM cell sensitivity to treatment and metabolism has been demonstrated in several studies^[63,64]^. Specific metabolic alterations may occur naturally (IDH mutation for example), while others occur as adapting processes during the acquisition of resistance^[64]^.

ROS production is instrumental in cell death induced either by TMZ or radiotherapy^[65]^. The ability of the cells to resist to TMZ treatment depends on the endogenous capacity of the cells to maintain a redox homeostasis^[66]^. The antioxidant apparatus and endogenous levels of ROS in the GBM cells, therefore, condition their ability to resist to treatment^[65]^. A study by Lo Dico *et al*.^[67]^ showed that TMZ caused fluctuations in cytoplasmic ROS levels inducing cytotoxic effects in TMZ-sensitive GBM cells while in TMZ-resistant GBM cells, no increase in cytoplasmic ROS levels were observed thus preventing cytotoxicity.

As GBM cells largely depend on glycolysis for their growth, several attempts have been made to inhibit glycolysis and induce cell death, by using 2-deoxy-D-glucose (2DG), 3-bromopyruvate or dichloroacetate (DCA)^[68-71]^. These strategies demonstrated a modest effect by when used individually but was more efficient as an adjuvant therapy to radio- or chemotherapy.

The acquisition of resistance relies on a transitory drug-tolerant cell population, related to the cancer stem-like cells, often exhibiting stem-like characteristics^[16]^. These slow-dividing, CD133+ cells demonstrate the highest dependency on glucose and are unable to shift their metabolism toward the use of glutamine during glucose deprivation^[72]^. Certainly, the dependence on glucose is higher in GCSC than in neural stem cells^[69]^, suggesting that glucose metabolism may be an interesting target for GBM cancer stem cells (GCSC). In this endeavor, DCA would be a better candidate than 2DG since 2DG inhibits the stem cell characteristics of both neural and GCSC^[69]^. Indeed, low-doses DCA induce a shift of GCSC toward oxidative metabolism, although no ROS production. This was sufficient to induce the loss of some stem cells characteristics, including the initiating cell capacity^[69]^, stem cell marker expression and triggered the induction of the expression of differentiated cell markers^[70]^. This resulted in an increase in the response to chemotherapy by GBM, through the p53-dependent on BH3-only proteins^[69]^ and the cytosolic sequestration and inactivation of Oct4 by PKM2^[70]^.

Aside from glycolysis, slow-cycling GCSC also rely on the oxidation of fatty acids for their survival^[73]^. Inhibiting fatty acid oxidation (for example with etoxomir^[73]^) is thus a possible alternative metabolic adjuvant strategy to overcome the resistance of GBM cells to therapy.

### The role of microenvironment in TMZ resistance

Tumorigenesis is a complex and dynamic process, which involves different cellular and non-cellular elements in the tumor microenvironment (TME). The interaction of the TME with cancer cells is responsible for tumor development, progression, and drug resistance. TME consists of non-malignant cells present in the tumor mass including cancer associated fibroblasts, endothelial cells and pericytes composing the tumor vasculature, immune and inflammatory cells, bone marrow-derived cells; and the extracellular matrix establishing a complex cross-talk within tumor mass. Tumor cells exclude extracellular vesicles (EV) to engage non-tumor cells in the TME and reprogram these cells from their normal activity to a more pro-tumorigenic. These EV contain and transport protein and nucleic acid cargoes to the non-tumor cells resulting in molecular, transcriptional and translational modifications that cause these cells to fabricate factors required for tumor growth and at the same time, alters the function of these cells. These cells could in turn generate their own EV containing and transferring molecules not only to the tumor but also to other cells in the TME enhancing their pro-tumorigenic activity. The EV represent a heterogeneous population of vesicles that can be divided into three large groups.

Exosomes are the smallest subset (50-100 nm) originating from the endocytic compartment of cells through a series of intraluminal invaginations occurring in multi-vesicular bodies.

Microvesicles are larger than exosomes (500-1000 nm) and are formed cell surface membrane blebbing and contain a random assortment of cell content.

Apoptotic bodies (800-5000 nm) represent cellular remains after apoptosis, containing an array of cellular debris.

Tumor-derived exosomes also contribute to the development of drug resistance. This can be achieved either by concentrating and removing the drug from the cytoplasm by exosomes or by packaging into exosomes to protect cells from the cytotoxic effects. Several studies have shown that drug resistance could be partially attributable to the intercellular transfer by exosomes of transporter proteins^[74,75]^ or miRNA^[76,77]^ from drug-resistant cells to sensitive cells.

An important step in anticancer treatment is the identification of the biological alterations present in TME to target these key molecular players. Multi-targeted approaches that providing a simultaneous inhibition of TME components have been shown to offer a more efficient way to treat certain cancers.

### Immunotherapy and TMZ

Clinical trials in GBM with checkpoint inhibitors and vaccination strategies have been so far very disappointing, probably because of the highly immunosuppressive environment of GBM. Another reason could be that most of these trials have targeted single components of an anti-tumor immune response without considering the heterogeneity of the GBM^[78]^. We recently showed that GBM cells with a mesenchymal signature are spontaneously eliminated by allogeneic human Vγ9Vδ2 T lymphocytes, through the cellular stress associated NKG2D pathway while other GBM subtypes were exempted from such reactivity^[79]^.

The relationship between TMZ and immune response in GBM has not been extensively studied^[80]^. However, contradictory TMZ immune-modulating effects have been reported and seem to depend on its time and the mode of delivery of the dose of TMZ^[81,82]^. On the other hand, quite promising results have shown that adjuvant immunotherapy against specific antigens, can efficiently eliminate TMZ-resistant GBM^[83,84]^. It is thus obvious that the immunotherapy regimen should be considered in combination with the effect of TMZ on the immune system^[85]^.

Resident brain macrophages and microglia are the main innate immune against central nervous system pathogens and insults. Indeed, these cells are an important component of GBM and may constitute up to 30%-50% of the total cell populations^[86]^. However, despite many recent advances, there are still numerous questions that remain to be answered about the identity, molecular drivers of recruitment, cancer induced reprogramming, polarization strategies and therapeutic modulation of GBM-associated macrophage and microglia immune biology.

In conclusion, the implication of the mechanisms of therapy resistance in the mutational burden, immunosuppression, and local immune dysfunction has still to be fully investigated in the context of combination therapy in a more personalized treatment in GBM.

### Persisters, stem cells and quiescence: the slow cycling connection

Since the original finding of slow cycling cells with stemness markers and high levels of DNA repair activity^[87]^, GCSC have been extensively studied. The role of GCSC in the resistance to therapy has been described in this review, as well as by other groups^[88]^. One of the current major questions regarding GCSC is the function of niche in their maintenance and resistance to treatment^[89]^. However, it seems that several types of GCSC can be found which are related to the different subtypes of GBM^[90]^. The characteristics of different GCSC subtypes remain to be identified to develop potential efficient and specific therapies. However, one well-known and common characteristic of GCSC is their slow cycling and quiescence activity. Indeed, several other types of cancer cells exhibit similar low proliferative activities and thus may represent new targets in cancer^[16]^. The existence of a class of drug tolerance with characteristics like persister cells described above has been recently identified in GBM. The work from the laboratory of Engelman showed that GCSC could reversibly transit to a slow-cycling, persister-like state in response to tyrosine kinase inhibitors (TKI)^[41]^. More interestingly, this adaptation to TKI was due to chromatin changes linked to an increased activity of histone demethylases KDM6A/B. Similarly, Banelli *et al*.^[40]^ identified histone demethylases as targets to overcome TMZ resistance. A transcriptomic study of the development of resistance to TMZ in the GBM cell line U251 identified a transient persister-like stage during which the cells were sensitive to histone deacetylase inhibitors^[91]^. In lung cancer cell line, Guler *et al*.^[14]^ showed that survival of persister cells was controlled by H3K9me3-mediated heterochromatin formation and that the disruption of the repressive chromatin over LINE-1 elements resulted in their eradication. Thus, chromatin states and its evolution under treatment might represent a new biomarkers and target in TMZ resistance in GBM.

The drug-tolerant cells appear also to be associate with a particular metabolic state with a marked increase in mitochondrial oxidative phosphorylation^[91]^, a characteristic of slow cycling cells in GBM^[92]^.

## Conclusion: is targeting persisters a future therapeutic option?

The heterogeneity of cancer cell populations increases with treatment-induced stress, causing resistance to emerge^[5]^. This is probably the main reason there are a multitude of mechanisms implicated in TMZ resistance. These mechanisms operate at different levels and probably cooperate within the tumor. It is thus difficult to envisage a treatment, which will target all mechanisms at the same time.

**Figure 4:**
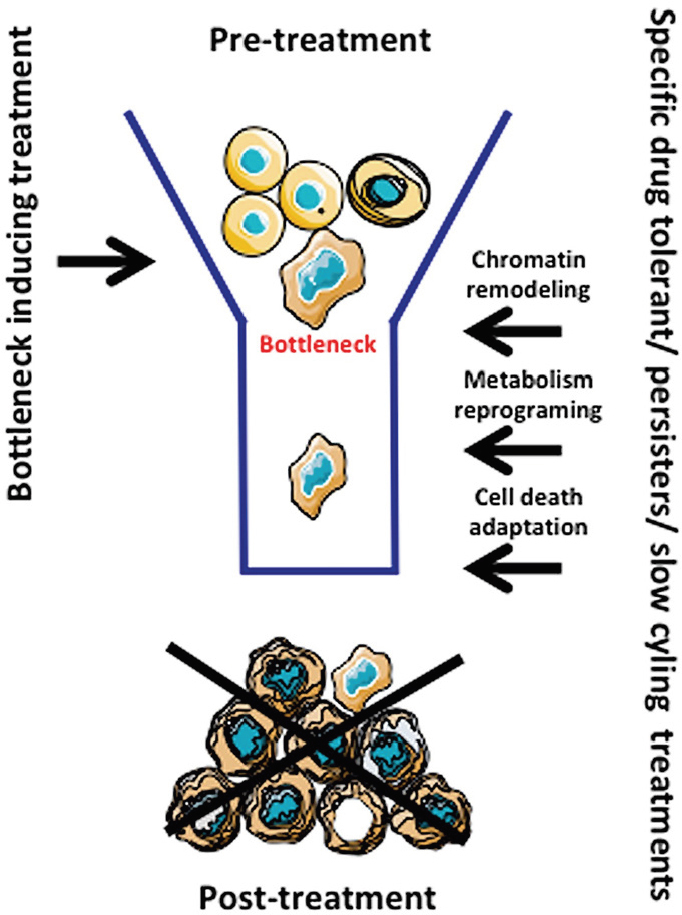
Formation of the bottleneck and resistance by treatment. The treatment (i.e., TMZ in GBM) induces a population restriction (bottleneck) and elicits an evolution/adaptation to treatment through cellular reprogramming. TMZ: Temozolomide; GBM: Glioblastoma

From the discussions above we can propose that a reduction in cellular heterogeneity and the elimination of persisters, which are the precursors of drug resistance, might be interesting strategies. The first response associated with the stress induced by treatment is a reduction in the population and heterogeneity of the cells leading to a “bottleneck” situation. This is followed by the emergence of a resistant cell population, which is also highly heterogeneous. An eradication strategy in GBM would thus involve sequential strategies: the first treatment should homogenize the population via adaptation to the induced stress, and the second treatment would specifically target the resulting bottleneck population. The literature points out a few dominant processes linked to this drug tolerant stage: chromatin remodeling, metabolism reprogramming and cell death adaptation [Figure 4]. It is noteworthy that therapies targeting these different mechanisms exist and could be easily evaluated in preclinical studies.

In some cases, a dominant resistant clone emerges, which could be sensitive to a second line of treatment. Recently, it appeared that resistance is linked to persisters, which undergo extensive reprogramming upon treatment. The resistant population might exhibit a few dominant clones, which in turn could be targeted by a combination of therapies. However, these resistant populations are often highly heterogeneous, impeding treatments; thus, targeting the persister population could efficiently reduce the therapeutic tools required and prevent the apparition of resistance.

## Declarations

### Copyright

© The Author(s) 2020.
